# Estimation and Human Health Risk Assessment of Organochlorine Pesticides in Raw Milk Marketed in Zagazig City, Egypt

**DOI:** 10.1155/2018/3821797

**Published:** 2018-08-12

**Authors:** Amal A. Raslan, Seham Elbadry, Wageh Sobhy Darwish

**Affiliations:** ^1^Educational Veterinary Hospital, Faculty of Veterinary Medicine, Zagazig University, 44519, Egypt; ^2^Food Control Department, Faculty of Veterinary Medicine, Zagazig University, 44519, Egypt

## Abstract

Milk is nearly a perfect natural food and is widely used by all segments of our population especially for infants and the elderly. Organochlorine pesticides (OCPs) have been used worldwide, particularly in many African countries as in Egypt for the control of pests. OCPs are characterized by their bioaccumulation in the environment, especially in the food chain, where they find their way into the human body. The objectives of this study were initially to estimate the residual concentrations of different OCPs in three kinds of fresh and raw milk from different animals (cattle, buffalo, and goat) marketed in Egypt. Additionally, human dietary intake and risk assessment of OCPs were calculated. The tested OCPs included pp-DDT and its metabolites pp-DDD and pp-DDE; hexachlorohexanes (HCHs) including *α* HCH and *γ* HCH; heptachlor and heptachlor epoxide; aldrin and endrin; chlordane, methoxychlor, and hexachloride benzene. The recorded results revealed that goat and buffalo milk samples had the highest incidence of OCPs' contamination (75% for each), while this percentage was 50% in cow's milk. The mean values of ΣOCPs were 317.83 ± 34.11, 605 ± 50.54, and 1210.57 ± 99.55 (ppb/ww) in the examined cattle, buffalo, and goat milk samples, respectively. All examined OCPs were within the maximum permissible limits (MPLs) set by World Health Organization with only 10% of goat milk samples exceeding this MRL. The estimated daily intake, noncancer, and cancer health risk assessment of the tested OCPs revealed the potential cancer risk especially among children consuming goat's milk. The public health importance of such OCPs was discussed.

## 1. Introduction

Milk is a complex, bioactive substance to promote growth and development of infant mammals. Cow, buffalo, and goat milk are widely consumed around the world, especially in Egypt. In fact, milk is considered as an ideal source of macroelements such as calcium, phosphorus, and potassium [1].

The widespread occurrence of any foreign chemical in the environment is a matter of public health concern. Pesticides are extensively used to increase agricultural products through preventing losses due to agricultural pests. The health authorities also use these chemicals to control various vectors, which spread diseases like malaria and plague [2].

Among the major groups of pesticides, organochlorines are more potent due to their persistence and stability. Universally important organochlorine pesticides (OCPs) are para, para, dichlorodiphenyltrichloroethane (pp-DDT), hexachloride benzene (HCB), chlordane, heptachlor, aldrin, dieldrin, and endrin. Due to the lipophilic nature of these pesticides, milk and other fat-rich substances are the key items for their accumulation [3]. These toxicants get into the human body through the food chain and cause serious health hazards [4].

Egypt as one of the most populous countries in Africa depend mainly on agricultural activities as major sources of national income. Therefore, pesticides are frequently used in Egypt to control pests or directly spread into animal skin for prevention and control of external parasites. These chemicals may find their way into animal body and subsequently pass into milk causing several toxicological implications for both animal and human if contaminated milk or other dairy products were consumed [5]. Studies had been done to investigate OCPs residues in different kinds of food including milk and other dairy products worldwide. However, in Egypt, few reports had surveyed the residual levels of OCPs in milk. In addition, the dietary intake and human health risk assessment due to consumption of the contaminated milk in Egypt is less informed.

Due to the previous facts, this study was conducted to firstly investigate the residual concentrations of OCPs in the milk of cattle, buffalo, and goat in Egypt. The tested OCPs included pp-DDT and its metabolites pp-DDD and pp-DDE; hexachlorohexanes (HCHs) including *α* HCH and *γ* HCH; heptachlor and heptachlor epoxide; aldrin and endrin; chlordane, methoxychlor, and HCB. Secondly, the dietary intake, carcinogenic, and noncarcinogenic risks due to consumption of such contaminated milk were calculated.

## 2. Materials and Methods

All experiments were done according to the rules and guidelines of Zagazig University, Egypt.

### 2.1. Sampling

Sixty milk samples (20 each of cow, buffalo, and goat milk) were randomly purchased from markets in Zagazig city, Sharkia province, Egypt. Raw milk is sold in Egypt in polyethylene bags, and each sample weighs 500 g. Samples were transferred into laboratory in a cooled container. Organochlorine pesticides were extracted and measured at Agricultural Research Center, Dokki, Giza, Egypt.

### 2.2. Detection of Organochlorine Compounds

#### 2.2.1. Chemicals

Standard OCPs including pp-DDT, pp-DDD, pp-DDE, *α* HCH, *γ* HCH, heptachlor, heptachlor epoxide, aldrin, endrin, chlordane, methoxychlor, and HCB were obtained from Sigma–Aldrich (Germany). Petroleum ether, diethyl ether, n-hexane, acetonitrile, anhydrous sodium sulfate, and methylene chloride were bought from Merck (Darmstadt, Germany). Florisil (PR Grade, 60–100 mesh) was purchased from Silica (Silica Co., USA). All solvents were of pesticide residue grade and subjected to a solvent purity test for residue analysis suitability. Florisil was activated at 130°C for 24 h and cooled to room temperature.

#### 2.2.2. Extraction and Preparation of Samples

Each individual sample (50 ml) was mixed with anhydrous sodium sulfate (100 g) and petroleum ether (150, 100, and 100 ml, respectively) in three successive extraction steps for 2 min each, as described before [6]. Anhydrous sodium sulfate removes water and helps to disintegrate the sample. Samples were filtered with a vacuum pump after each extraction. The solvent was evaporated on a rotary evaporator at 40°C until dryness.

#### 2.2.3. Partitioning of the Extract

Partitioning of the extracted samples was carried out according to the method of the Association of Official Analytical Chemists [7]. At first 500 ml n-hexane was partitioned with an equal volume of acetonitrile by mixing these two solvents in a separating funnel followed by separation of each solvent to be used for sample partitioning. The extracted sample was transferred with a mixture of 80 ml n-hexane and 20 ml acetonitrile into a 100-ml separating funnel, followed by vigorous shaking for 2 min. After separation of two solvent layers, acetonitrile was collected in a flask after being passed through anhydrous sodium sulfate to remove any moisture. Another 20 ml acetonitrile was added to n-hexane and the aforementioned partitioning step was repeated 3 times. Finally, n-hexane was discarded while acetonitrile was evaporated on a rotary evaporator to a volume less than 10 ml to be used for florisil cleanup.

#### 2.2.4. Cleanup of the Extract

Cleanup of the extracted samples, to remove the residual fat, was performed by transferring the extract into a glass chromatographic column (22 mm i.d.) containing 20 g activated florisil (60–100 mesh) topped with 1-cm layer of anhydrous sodium sulfate. The prepared column was firstly rinsed with 50 ml petroleum ether, and then the extracted sample was transferred onto the column. The column was eluted with 200 ml eluent (10% anhydrous diethyl ether + 90% petroleum ether) followed by a second elution with 100 ml of another eluent (1% acetonitrile + 29% n-hexane + 70% methylene chloride). The collected eluent was concentrated on a rotary evaporator and dissolved in hexane to a volume of 10 ml. An aliquot of each extract was transferred to 2-ml injection vials to be ready for the analysis with the electron capture gas chromatography.

#### 2.2.5. Determination of Organochlorine Pesticide Residual Concentrations

Organochlorine residues were determined by analysis of samples using electron capture gas chromatography (Hewlett Packard GC Model 6890) equipped with Ni63–electron capture detector. GC conditions were HP- 5MS capillary column (30m length X 0.32mm internal diameter (i.d..), X 0.25*μ*m film thickness; carrier gas: N_2_ at a flow rate of 4 ml/min; injector and detector temperatures were 230°C and 300°C, respectively). The extract was injected into a single inlet that was split into the dual columns. Instrumental settings were as follows: injector and detector temperatures were 230°C and 300°C, respectively; the gas chromatography oven temperature program was initiated at 150°C for 5 min, raised to 170°C (at a rate of 5°C/min) and held for 10 min, then raised to 220°C (at a rate of 10°C/min) and held for 20 min (with a total run time of 44 min); the injection volume was 1, ll, and the flow rates of nitrogen make-up gas were 20 ml/min.

#### 2.2.6. Quality Assurance of Analytical Procedures

Calibration standard curves were created and the organochlorine pesticide residues were quantitatively determined by comparison with the standard solutions injected under the identical gas chromatography conditions. The standard reference material, SRM 1947 (Lake Michigan Fish Tissue), was analyzed during the analysis of samples followed by the same procedure of extraction, cleanup, and analysis. The percentage of recoveries of the organochlorines tested ranged from 86% to 109%. Residue levels for each pesticide were subsequently corrected for the recovery values. The limits of detection (LOD) and quantification (LOQ) for the tested OCPs were based on 3:1 signal to noise ratio (S/N) and ranged from 0.004 to 0.20 ng g^−1^ (LOD) and 0.024 to 0.036 ng g^−1^ (LOQ).

### 2.3. Human Health Risk Assessment

To estimate human health risks due to ingestion of OCPs contaminated milk among Egyptian populations (children and adults), both estimated daily intake (EDI) and hazard ratio (HR) were calculated based on the equations recommended by USEPA [8].

(1) $$\begin{array}{c} EDI=C*\frac{Fi}{Bwt} \end{array}$$

where C is the average concentration of the chemical contaminants (ng g/ww) in the milk. Fi is the average daily intake, based on the information retrieved from Egyptian consumers; Fi was set to be 200 g and 400 g for adults and children, respectively. Bwt is the average body weight for Egyptian adults (60 kg) and children (30 kg) [9]. EDIs were compared with the acceptable daily intake (ADI) [10].

Noncancer and cancer risk assessment were calculated using hazard ratio (HR). A hazard ratio higher than one indicates potential human health risks [11].

(2) $$\begin{array}{c} HRs=\frac{EDI}{BMC} \end{array}$$

The benchmark concentration (BMC) for carcinogenic effects was derived from cancer slope factor (CSF) and for noncarcinogenic effects was based on the oral reference dose (RFD). Both CSF and RFD were obtained from the United States Environmental Protection Agency Integrated Risk Information System [8].

### 2.4. Statistical Analysis

All values are expressed as means ± SE, and all measurements were carried out in duplicate. Statistical significance was evaluated using the comparative of means method (the Tukey–Kramer HSD test) (JMP statistical package; SAS Institute Inc., Cary, NC).

## 3. Results and Discussion

Organochlorine pesticides (OCPs) have been used worldwide, particularly in Africa for several decades. Although many are banned, several African countries still use OCPs especially for the prevention and control of malaria. OCPs are characterized by their bioaccumulation in the environment, especially in the food chain, where they find their way into the human body.

### 3.1. Residue Levels of OCPs in Marketed Milk in Egypt

In this study, the residual concentrations of different OCPs in three kinds of marketed milk (cattle, buffalo, and goat) in Egypt were estimated.

**Figure 1:**
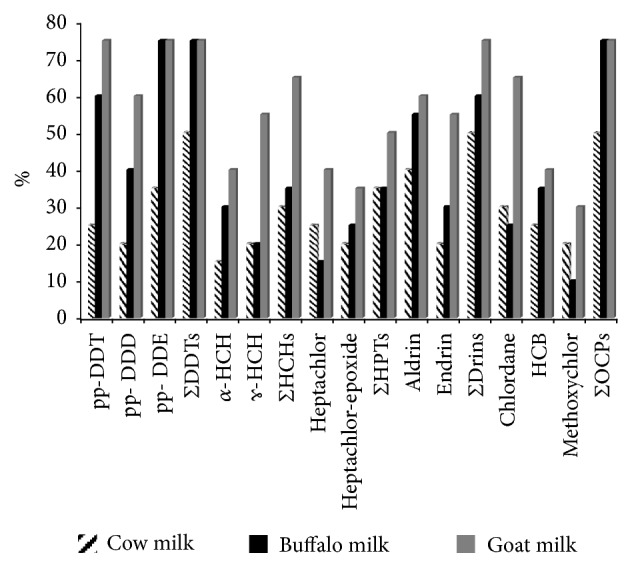
Frequency (%) of individual and total OCPs contamination of the examined milk samples from different animal species (n=20 each).

The recorded results revealed that goat and buffalo milk samples had the highest contamination level of OCPs (75%, 15 out of 20 examined samples), while this percentage was 50% (10 out of 20 examined samples) in cow's milk (see Figure 1). OCP-positive samples in the current investigation was lower than those detected by Heck* et al.* [12], who reported positive frequencies (100%) in the examined buffalo and sheep milk samples marketed in Brazil.

**Figure 2:**
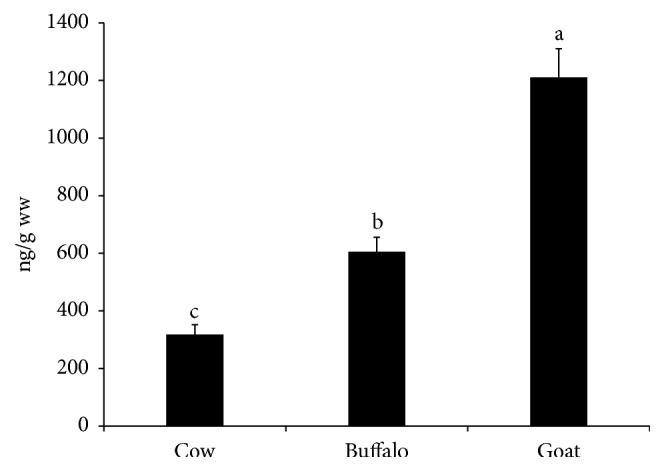
**Total organochlorine pesticide residues (OCPs) in the examined milk samples.** Data represent mean ± SE (ng/g ww) for total OCPs in the examined milk samples from different animal species (n=20 each). Columns that carry different superscript letter are significantly different at p<0.05.

The mean values of ΣOCPs in the examined milk samples were 317.83 ± 34.11, 605 ± 50.54, and 1210.57 ± 99.55 (ng/g ww) in the examined cattle, buffalo, and goat milk samples, respectively (see Figure 2). The recorded concentrations in this study were much lower than the concentrations recorded in buffalo milk by in India (8571 ng/g ww) [13]. However, these concentrations were comparable to 874.40 and 485.76 (ng/g ww) that reported in goat's milk and cheese retailed in Ethiopia and Ghana, respectively [14, 15].

**Figure 3:**
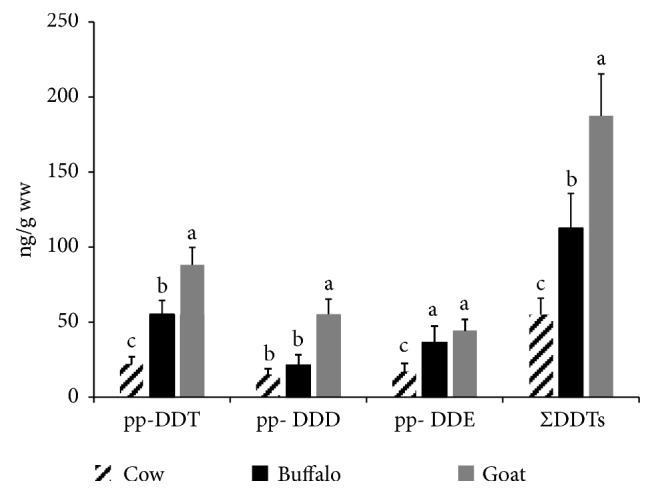
**Total DDT and its metabolites in the examined milk samples.** Data represent mean ± SE (ng/g ww) for total DDT and its metabolites in the examined milk samples from different animal species (n=20 each). Columns that carry different superscript letter among the same chemical are significantly different at* p*<0.05.

Although OCPs' use has been banned in Egypt since the 1980s, DDTs are still detected in various foods in the country. For instance, mussels from Abu Qir Bay contained several OCPs, with DDT concentrations up to 31000 (ng/g dw), but a risk assessment showed no expected adverse effects on people through mussel consumption [16]. In the present study, either pp-DDT or its metabolites pp-DDD and pp-DDE were detected in 50%, 75%, and 75% of the examined cattle, buffalo, and goat milk samples, respectively (see Figure 1). The frequency of detection of OCPs in this study was comparable to that detected in raw milk from other countries (35-75%) as in China, India, Mexico, and Slovakia [13, 17–19]. The recorded residual concentrations of the detected DDTs were graphed (see Figure 3). Goat milk had significantly (*p*< 0.05) the highest ΣDDTs followed by buffalo and cattle milk samples. The average concentrations were 187.49 ± 27.88, 112.66 ± 23.11, and 54.77 ± 11.14 (ng/g ww) in the goat, buffalo, and cattle milk samples, respectively (see Figure 3). These results also show that the detected concentrations of either pp-DDT or its metabolites pp-DDE and pp-DDD were low, when compared with the established maximum residual concentrations (MRLs) (200 ng/g ww) by World Health Organization [20]. Presence of residues of DDTs in the milk samples indicate the past use of these pesticides in the agricultural activities in Egypt. In correspondence to the results of this study, Darko and Acquaah [14] detected DDTs in milk, yoghurt, and cheese marketed in Ghana in concentrations ranged from 0.01 to 119 ng/g ww. Unlikely, higher concentrations of DDTs (1230 and 874.4 ng/g ww) were recorded in cattle and goat milk samples collected from Ethiopian markets [15]. In contrast, Shaker and Elsharkawy [21] did not detect DDTs in the buffalo milk samples collected from Assuit city, Egypt.

**Figure 4:**
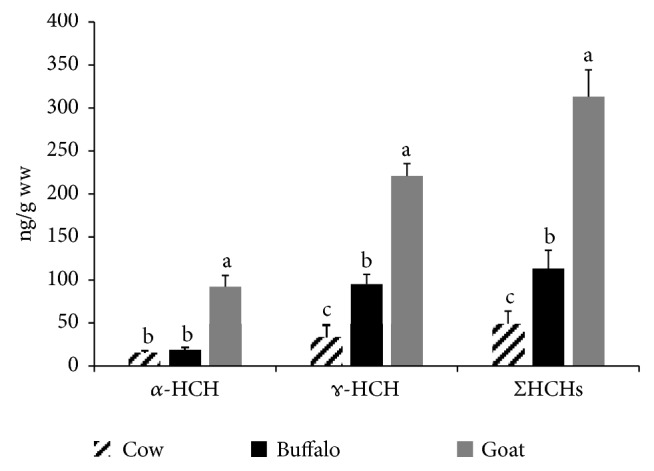
**Total HCH and its isomers' residues in the examined milk samples.** Data represent mean ± SE (ng/g ww) for total HCH and its isomers in the examined milk samples from different animal species (n=20 each). Columns that carry different superscript letter among the same chemical are significantly different at* p*<0.05.

Hexachlorocyclohexanes (HCHs) were detected in 30%, 35%, and 65% of the examined cattle, buffalo, and goat milk samples, respectively (see Figure 1). Data presented in Figure 4 represent ΣHCHs and its *α*-HCH and *ɤ*-HCH isomers. The average ΣHCHs values were 48.65 ± 15.12, 113.27 ± 21.23, and 313.16 ± 31.11 (ng/g ww) in the examined cattle, buffalo, and goat milk samples, respectively. Lindane (*ɤ*-HCH) is the most active and stable isomer of HCHs. The residual concentrations of lindane in the examined milk samples were 34.44 ± 14.1, 95.11 ± 11.33, and 221.00 ± 14.25 (ng/g ww) in cattle, buffalo, and goat milk samples, respectively. It is clear that goat had significantly the highest *α*-HCH, *ɤ*-HCH, and ΣHCHs followed by buffalo and finally cattle samples (see Figure 4). Codex Alimentarius Commission set MRLs of lindane to be 200 (ng/g ww) [22], with only 10% (2 out of 20) of goat milk samples exceeding this MRL. The recorded concentrations in this study go in agreement with the detected concentrations of ΣHCHs in buffalo liver, kidney, and tongue (34.97-351.57 ng/g lw) collected from Zagazig slaughter house [23].

Furthermore, the concentrations of HCHs in this study were comparable to the recorded concentrations in cattle raw milk marketed in Egypt, India, Ghana, Mexico, and Uganda [13, 14, 18, 21, 24].

**Figure 5:**
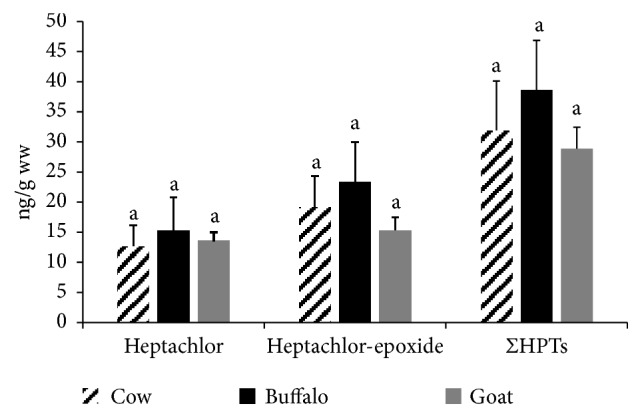
**Total heptachlor and its epoxide metabolite in the examined milk samples.** Data represent mean ± SE (ng/g ww) for total heptachlor and its epoxide in the examined milk samples from different animal species (n=20 each). Columns that carry same superscript letter among the same chemical are not significantly different at* p*<0.05.

Heptachlor and its epoxide were detected in 35%, 35%, and 50% of the examined cattle, buffalo, and goat milk samples, respectively (see Figure 1). The sum values of heptachlor and its metabolite were 31.88 ± 8.23, 38.63 ± 8.22, and 28.88 ± 3.56 (ng/g ww) in the examined cattle, buffalo, and goat milk samples with no significant differences among examined species (see Figure 5). The recorded concentrations of Σheptachlors in the current study were lower than that reported in buffalo milk in India (335 ng/g ww) [13]. None of the examined samples in the present study exceeded MPLs of heptachlors (150 ppb) [20].

**Figure 6:**
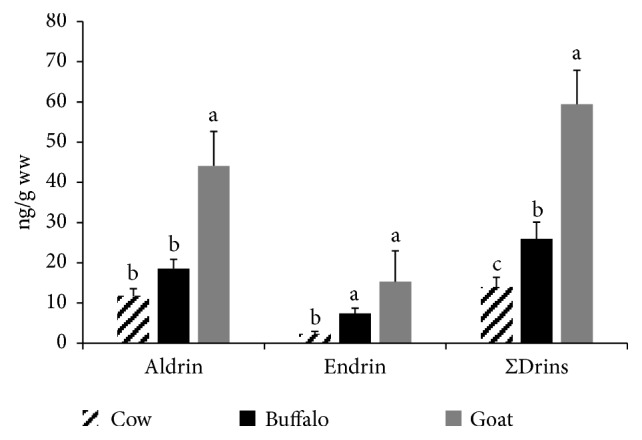
**Total drins in the examined milk samples.** Data represent mean ± SE (ng/g ww) for total drins in the examined milk samples from different animal species (n=20 each). Columns that carry same superscript letter among the same chemical are not significantly different at* p*<0.05.

Drins either aldrin or endrin were detected in 50%, 60%, and 75% of the examined cattle, buffalo, and goat milk samples, respectively (see Figure 1). The mean residual concentrations of the total drins were 13.96 ± 2.44, 25.95 ± 4.16, and 59.43 ± 8.44 (ng/g ww) in the examined cattle, buffalo, and goat milk samples with the goat milk in the top of the examined species (see Figure 6). All examined samples were within total drins' MPL (150 ppb) [20]. The concentrations of the Σdrins in buffalo's milk in the present study were comparable to the recorded values in fresh buffalo's milk in Egypt (15 ng/g ww) and in cheese in Ghana (7.88 ng/g ww) [14, 21].

**Figure 7:**
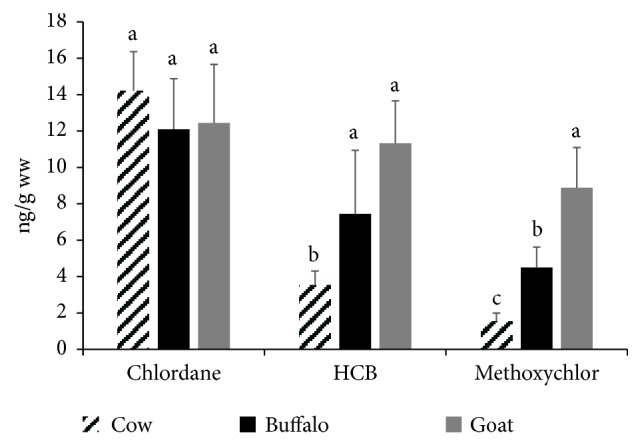
**Chlordane, HCB, and methoxychlor residues in the examined milk samples.** Data represent mean ± SE (ng/g ww) for chlordane, HCB, and methoxychlor residues in the examined milk samples from different animal species (n=20 each). Columns that carry same superscript letter among the same chemical are not significantly different at* p*<0.05.

Other examined OCPs such as chlordane, HCB, and methoxychlor were detected in 10-60% (see Figure 1). The positive samples had minute concentrations of these OCPs that ranged from 1.55 to 14.21 (ng/g ww) (see Figure 7); all samples were below MPLs [20]. Nearly, similar values were reported in Assiut, Egypt [21].

 It is worth noting that in the present work goat milk had the highest OCPs residues. This may be due to the grazing behavior of the goat. Additionally, buffalo's milk had higher OCPs compared with the milk of the cow. This may be attributed either to the high fat content (7.47%) of the buffalo's milk or to the dietary habits of the buffalo, like different fodder and to variations in diets compared with the cows [13].

### 3.2. Human Dietary Intake and Risk Assessment of OCPs

Humans can be exposed to OCPs via several routes including breathing of polluted air, dermal penetration, or ingestion of contaminated foods and drinking water. OCP-contaminated foods like milk and other dairy products are considered the main source of human exposure to pesticides [25]. In the current study, EDIs of different OCPs were presented in Table 1. In general, the calculated EDIs were far below the acceptable daily intakes [10]. However, among the analyzed samples, consumption of goat's milk especially by children is alarming for heptachlors and drins. The low dietary intake of other OCPs may be due to the restriction of the use of OCPs in the agricultural activities. In correspondence with EDIs of OCPs in milk, Mahmoud* et al.* [26] reported relatively similar EDIs for OCPs via consumption of meat and offal marketed in Egypt.

The analyzed OCPs in the present study had both cancer and noncancer risks [27]. Cancer and noncancer hazard ratios through consumption of milk in Egypt for both adults and children were summarized in Tables 2 and 3. Noncancer HR values were far below one in all analyzed OCPs except for methoxychlor (see Table 2). However, the lifetime cancer risks were considered high in the present study, especially for DDTs and HCHs among children consuming goat's milk (see Table 3). Similarly, cancer HR greater than one were reported in studies conducted in Egypt and Mexico [26, 28].

Maternal transfer is also possible across the placenta to the foetus or via breast milk to infants. Residue levels of these compounds in living organisms depend on each organism's habitat and position in the food chain [29]. OCPs are classified among the endocrine disrupting chemicals [30], which are linked to several toxicological implications that include reduced fertility, spontaneous abortion [31], and reproductive tract anomalies among both sexes [32].

In conclusion, high concentration of the tested OCPs reveals the increased improper use of these pesticides by the farmers for agricultural purposes. These pollutants are characterized by long persistence in the environment and thus may pass to next generations of humans and different plant and animal species. Thus, continuous monitoring studies to investigate the status of OCPs contamination in the Egyptian environment and food subjects are mandatory in Egypt.

## Figures and Tables

**Table 1:** Estimated daily intake of OCPs due to ingestion of milk among Egyptian population.

	**ADI**	**Cow**'s** milk**		**Buffalo**'s** milk**		**Goat**'s** milk**	
		**Adults**	**Children**	**Adults**	**Children**	**Adults**	**Children**
**pp-DDT**	10000	74.03	296.13	184.43	737.73	293.7	1174.8

**pp-DDD**	10000	51.1	204.4	70.36	281.46	184.1	736.4

**pp-DDE**	10000	57.43	229.73	120.73	482.93	147.16	588.66

**Heptachlor**	100	42.23	168.93	50.76	203.06	45.16	180.66

**Heptachlor-epoxide**	100	64.03	256.13	78	312	51.1	204.4

**α** **-HCH**	5000	50.7	202.8	60.53	242.13	307.2	1228.8

**ɤ** **-HCH**	5000	111.46	445.86	317.03	1268.13	736.66	2946.66

**Aldrin**	100	38.86	**155.46**	61.83	**247.33**	**147.03**	**588.13**

**Endrin**	100	7.66	30.66	24.66	98.66	51.06	**204.26**

**Chlordane**	500	47.36	189.46	40.33	161.33	41.46	165.86

**HCB**	600	11.83	47.33	24.8	99.2	37.76	151.06

**Methoxychlor**	6300	5.16	20.66	15	60	29.6	118.4

ADI: acceptable daily intake.

Values in bold are higher than ADI.

**Table 2:** Noncancer hazard ratio among Egyptian population due to ingestion of OCPs-contaminated milk from different animal species.

	**RFD**	**Cow**'s** milk**		**Buffalo**'s** milk**		**Goat**'s** milk**	
		**Adults**	**Children**	**Adults**	**Children**	**Adults**	**Children**
**pp-DDT**	5.00E-04	0.04	0.15	0.09	0.37	0.15	0.59

**pp-DDD**	5.00E-04	0.03	0.1	0.04	0.14	0.09	0.37

**pp-DDE**	5.00E-04	0.03	0.11	0.06	0.24	0.07	0.29

**Heptachlor**	5.00E-04	0.02	0.08	0.03	0.1	0.02	0.09

**Heptachlor-epoxide**	5.00E-04	0.03	0.13	0.04	0.16	0.03	0.1

**α** **-HCH**	3.00E-04	0.02	0.06	0.02	0.07	0.09	0.37

**ɤ** **-HCH**	3.00E-04	0.03	0.13	0.09	0.38	0.22	0.88

**Aldrin**	3.00E-04	0.01	0.05	0.02	0.07	0.04	0.18

**Endrin**	3.00E-04	0.002	0.01	0.01	0.03	0.02	0.06

**Chlordane**	5.00E-04	0.02	0.09	0.02	0.08	0.02	0.08

**HCB**	8.00E-04	0.01	0.04	0.02	0.08	0.03	0.12

**Methoxychlor**	0.05	0.26	**1.03**	0.75	**3**	**1.48**	**5.92**

RFD: oral reference doses.

Values in bold represent higher hazard ratio (>1.0).

**Table 3:** Cancer hazard ratio among Egyptian population due to ingestion of OCPs-contaminated milk from different animal species.

	**CSF**	**Cow milk**		**Buffalo milk**		**Goat milk**	
		**Adults**	**Children**	**Adults**	**Children**	**Adults**	**Children**
**pp-DDT**	0.34	0.22	0.88	0.54	**2.17**	0.86	**3.46**

**pp-DDD**	0.24	0.21	0.85	0.29	**1.17**	0.77	**3.07**

**pp-DDE**	0.34	0.17	0.68	0.36	**1.42**	0.43	**1.73**

**Heptachlor**	4.5	0.01	0.04	0.01	0.05	0.01	0.05

**Heptachlor-epoxide**	4.5	0.01	0.06	0.02	0.07	0.01	0.05

**α** **-HCH**	1.1	0.05	0.18	0.06	0.22	0.28	**1.12**

**ɤ** **-HCH**	1.1	0.1	0.41	0.29	**1.15**	0.67	**2.68**

**Aldrin**	17	0.002	0.009	0.004	0.015	0.01	0.03

**Endrin**	17	0.0004	0.002	0.001	0.006	0.003	0.01

**Chlordane**	0.35	0.14	0.54	0.115	0.46	0.12	0.47

**HCB**	1.6	0.01	0.03	0.02	0.06	0.02	0.09

**Methoxychlor**	NA	NA	NA	NA	NA	NA	NA

CSF: cancer slope factor.

NA: not available as there is no CSF value for methoxychlor.

Values in bold represent higher hazard ratio (>1.0).

## Data Availability

The data used to support the findings of this study are available from the corresponding author upon request.
